# Theoretical analysis and experiment of pressure distribution and pressure gradient of shield screw conveyor: Taking sandy soil as an example

**DOI:** 10.1038/s41598-020-64254-3

**Published:** 2020-05-12

**Authors:** Xingchun Li, Yi Yang, Dalong Jin, Xinggao Li

**Affiliations:** 1 Department of Intelligent Manufacturing, Wuyi University, Guangdong, Jiangmen 529020 P.R. China; 2 School of Civil Engineering, Beijing Jiaotong University, Beijing, 100044 P.R. China

**Keywords:** Civil engineering, Mechanical engineering

## Abstract

The pressure distribution and pressure gradient characteristics of shield screw conveyor directly affect its normal function. Taking the conditioned soil microelements in the spiral tube as the research object, the pressure field distribution and pressure gradient theoretical calculation model of the shield screw machine were established based on the stress analysis and the mechanical equilibrium conditions at steady state and the influence parameters were analyzed. The correctness and validity of the proposed calculation model are verified by the experimental results of the indoor model machine. Under the condition that the conditioned soil is the plastic fluid paste, the conclusion that the pressure field and pressure gradient of the shield screw machine decrease linearly along the direction of the screw axis is also obtained.

## Introduction

**Figure 1:**
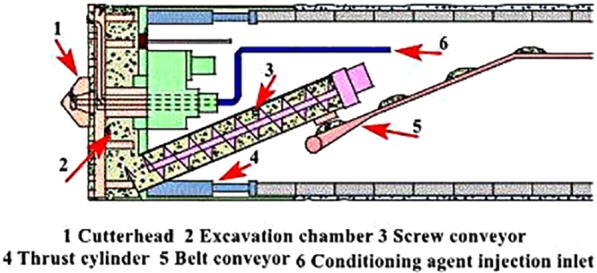
Schematic diagram of earth pressure balance shield machine.

The screw conveyor is an important component of the earth pressure balanced shield machine and bears the heavy responsibility of “soil discharge, pressure holding and pressure regulation”. Understanding the soil pressure distribution and pressure gradient in the earth pressure balanced shield screw conveyor is critical to regulating the earth chamber pressure and reducing ground disturbance. In case of shield tunneling in the high-pressure water-rich sandy layer, soil conditioning is inevitable. The pressure distribution and pressure gradient of the shield screw conveyor is heavily influenced by the mechanical properties of the conditioned soil together with the structural parameters of the shield screw conveyor. Knowing this pressure distribution and pressure gradient is of paramount importance to control soil blasting (water gushing) at the conveyor exit. So it is necessary to theoretically model the pressure distribution and pressure gradient of the shield screw conveyor. The establishment of the model of pressure distribution and pressure gradient of the shield screw conveyor will provide certain technical guidance for the soil conditioning strategy and the structural design of the shield screw conveyor. The main components of the earth pressure balanced shield machine and the soil removal function of the screw conveyor are shown in Fig. 1.

The researches on the working mechanism of general type screw conveyors for material transfer are earlier and have more results. For example, Darnell and Mol^1^, Chung^2^ established pressure gradient distribution theory model along the screw conveyor; Owen and Cleary^3^, Pezo *et al*.^4^ used the discrete element method to simulate the movement of material particles in the screw conveyor. Liu *et al*.^5^ created a motion model for the horizontal screw conveyor and optimized its parameters. Li^6^ proposed a structural optimization design method for mining screw conveyor based on Matlab.

The research on the pressure-holding mechanism of the shield screw conveyor has achieved relatively late results. Yoshikawa^7^ assumed that the soil is a friction material and a plastic material, and derived linear and nonlinear pressure gradient models distributed along the screw machine, respectively. Based on the assumption of plastic materials, Yoshikawa^8^ gave equations for calculating the pressure gradient of belt screw conveyors with different structures. Yoshikawa^9^ systematically expounded the influence of screw machine structure, size, operating conditions and soil conditioning on its working performance. Akimasa *et al*.^10^ proposed and verified the theoretical equation of the water stopping capacity of the earth pressure balanced shield screw machine. Bezuijen and Schamine´e^11^ based on the results of laboratory model tests, assuming that the shear stress at the interface between the soil and the screw machine is constant, and concluded that the pressure gradient is linearly distributed along the length of the screw machine. Talmon and Bezuijen^12^ assumed that the conditioned soil was a plastic slurry and the interface shear stress was constant, and a linear pressure gradient model distributed along the screw machine was proposed. Merritt^13^, Merritt and Mair^14^ conducted a soil discharge test of conditioned London clay using a screw excavator model machine, and tested the results of the linear distribution of total stress along the screw conveyor. Bezuijen *et al*.^15^ explained the pressure reduction mechanism of the shield screw machine based on the measured data of the project. Jiang *et al*.^16^ deduced the mechanical expression of the pressure at the bottom of the screw conveyor under static equilibrium conditions, and discussed its relationship with equipment parameters and soil properties. Jiang *et al*.^17^ established a mechanical expression of the pressure at the bottom of a double-cascading screw conveyor under static equilibrium conditions.

Duarte^18^ conducted a series of index evaluation tests of foam-conditioned sandy soil using a spiral excavator model. Peila *et al*.^19^ used the prototype experimental device of a spiral excavator to conduct a soil discharge test of foam-conditioned medium-sized sand. Merritt and Mair^20^ established a calculation model of the pressure gradient and its torque in the screw machine, and combined with the test results of the clay sample for verification analysis. Li^21^ assumed that the conditioned soil conformed to the non-Newtonian fluid Bingham model, and simulated the soil flow in the screw machine. Zhang^22^ ignored the influence of spiral blades on soil and derived the equation for calculating the maximum pressure drop of a two-stage screw conveyor. Kim *et al*.^23^ used model tests to study the effects of blade angle, pitch, and screw conveyor speed on the amount of soil discharge. Shangguan^24^ based on the Duncan-Chang nonlinear elastic constitutive model of conditioned soil in the sealed shield chamber, and established a mapping relationship between the pressure of the shield chamber and its advance speed and the screw rotation speed. Meng^25^ revealed the characteristics of the controlled system such as the pressure transmission characteristics of the shield screw machine and the effect of the control parameters on the pressure of the shield chamber. Zhou^26^ systematically analyzed the soil discharge and pressure holding mechanism of the earth pressure balanced shield screw machine. Li^27^ proposed the configuration and selection standard of the earth pressure balanced shield screw conveyor. Li^28^ proposed improvement ideas for the problems existing in the shield screw conveyor structure. Zheng *et al*.^29^ used the fluent software to carry out numerical simulation of particle flow in screw conveyor. Tao *et al*.^30^ analyzed the dynamic performance of the screw conveyor using the abaqus software. Zhang^31^ analyzed the pressure and body deformation of the soil flow field conveyed by the shield screw based on the CFD fluid-solid coupling software. Oh *et al*.^32^ used model tests to analyze the effects of the type of screw excavator (shaft type, belt type), blade angle, and mounting angle of the excavator on its working efficiency. Talebi *et al*.^33^ regarded the conditioned soil as Bingham liquid, used computational fluid dynamics to simulate the flow of the muck in the screw conveyor, and calculated the rheological parameters of the conditioned soil by back calculation. Zhang *et al*.^34^ used the DEM method to carry out research on the optimization of the structure of the dual-axis screw conveyor. Zhu and Tan^35^ used the EDEM software to analyze the effects of parameters such as rotation speed and pitch on screw conveyor performance.

Based on the above research results, assuming that the filling rate of the conditioned soil in the spiral tube is 100% and the conditioned soil is a plastic fluid paste, and the isotropic condition is used, the theoretical calculation equation of the pressure field distribution and pressure gradient of the conditioned soil in the spiral direction of the screw conveyor at steady state was given by the stress analysis of the soil microelement; Finally, the rationality and effectiveness of the proposed theoretical calculation model were verified by model machine laboratory tests, and the limitations of the theoretical calculation method were also pointed out.

## Pressure field distribution and pressure gradient mechanical model

### Mechanical equilibrium equation in the direction of the spiral axis

**Figure 2:**
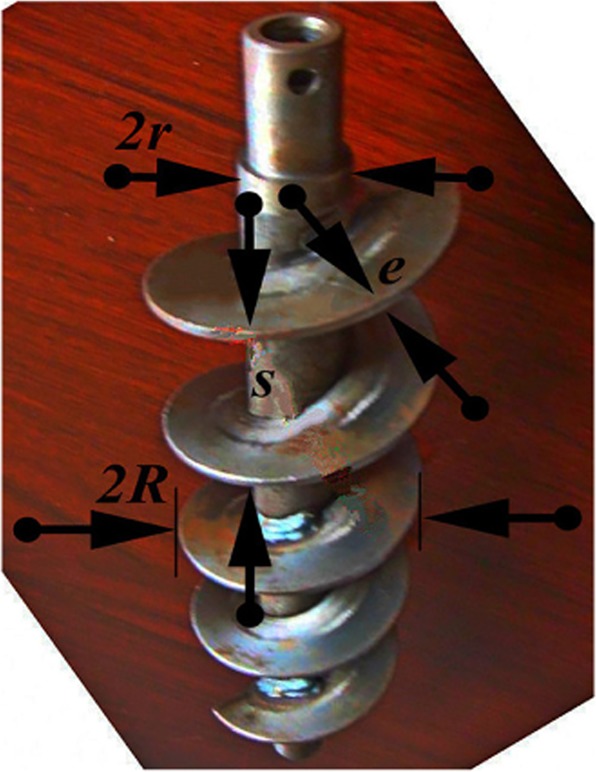
Screw structure.

The shield model screw structure size is shown in Fig. 2.

Where *e* is the thickness of the spiral blade, *s* is the pitch, *2r* is the diameter of the spiral shaft, and *2R* is the diameter of the spiral blade.

The helix angle *φ* is defined by Eq. (1).

1 $$\sin \,\varphi =\frac{dy}{dl}$$

Where d*y* is the axial micro-increment; d*l* is the micro-increment of the spiral direction of the spiral blade.

The definition of the spiral angle of the outer edge of the spiral blade is shown in Eq. (2), and the definition of the spiral angle at the spiral axis is shown in Eq. (3).

2 $$\varphi =\arctan \frac{s}{2\cdot \pi \cdot R}$$

3 $${\varphi }_{s}=\arctan \frac{s}{2\cdot \pi \cdot r}$$

**Figure 3:**
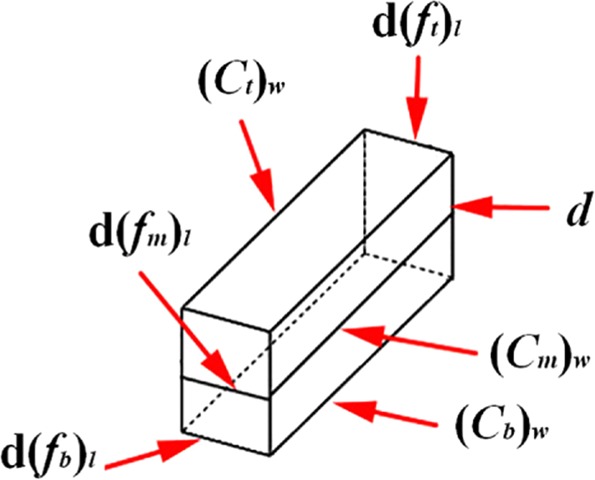
Dimensions of research object.

During the construction of a shield tunnel, the screw conveyor is used to transport the muck and the muck fills the spiral tube completely. Taking the soil microelement in the spiral tube as the research object, the length definition of the microelement is shown in Fig. 3.

Where the length of the top of the microelement body along the spiral groove direction is d(*f*_*t*_)_*l*_, the average length is d(*f*_*m*_)_*l*_, and the bottom length is d(*f*_*b*_)_*l*_; the top width of the microelement in the groove is (*c*_*t*_)_*w*_, the average width is (*c*_*m*_)_*w*_, the bottom width is (*c*_*b*_)_*w*_, and the groove depth is *d*. The parameters satisfy the relationship of Eqs. (4) to (10).

4 $$\sin \,\varphi ={\frac{dy}{d({f}_{t})}}_{l}$$

5 $$\sin \,{\varphi }_{m}={\frac{dy}{d({f}_{m})}}_{l}$$

6 $$\frac{d{({f}_{t})}_{l}}{d{({f}_{m})}_{l}}=\frac{R}{R-\frac{d}{2}}$$

7 $$\frac{d{({f}_{b})}_{l}}{d{({f}_{m})}_{l}}=\frac{r}{R-\frac{d}{2}}$$

8 $${({c}_{t})}_{w}=(s-e)\cdot \,\cos \,\varphi $$

9 $${({c}_{m})}_{w}=(s-e)\cdot {\rm{\cos }}{\varphi }_{m}$$

10 $${({c}_{b})}_{w}=(s-e)\cdot \,\cos \,{\varphi }_{s}$$

**Figure 4:**
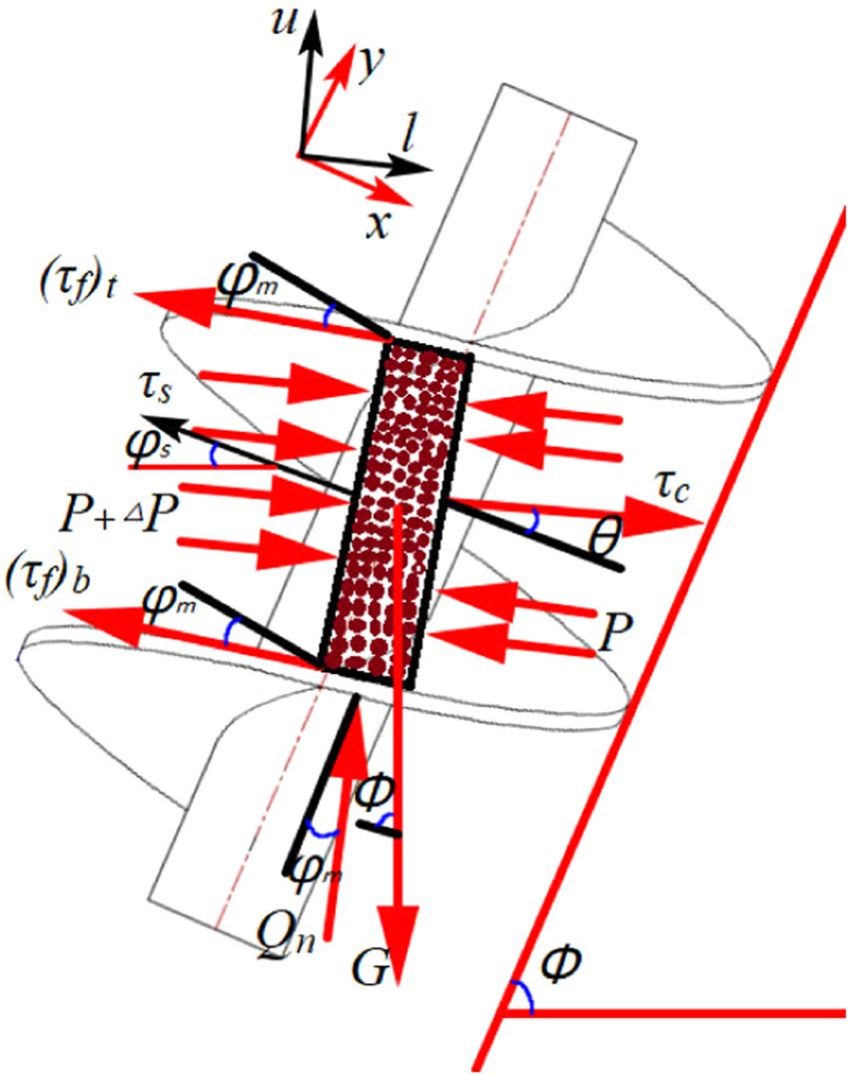
Stress analysis of the soil microelement.

The soil microelement in the spiral tube is subjected to stresses and force, which are the shear stresses (the upper spiral blade shear stress (*τ*_*f*_)_*t*_, the lower spiral blade shear stress (*τ*_*f*_)_*b*_, the spiral shaft shear stress *τ*_*s*_, and the spiral shell shear stress *τ*_*c*_), the combined compressive stress *Q*_*n*_ of the upper and lower spiral blades, the pressure *P* in the direction of the spiral groove, and the gravitational force *G* of the soil microelement. For the convenience of analysis, two coordinate systems are defined in the Fig., (*x, y*) are vertical and parallel spiral axis coordinate systems; (*l, u*) is the coordinate system of parallel and vertical spiral blades. The specific force situation is shown in Fig. 4.

Where *φ* is the installation leaning angle and θ the soil flow conveying angle, and the soil flow conveying angle is defined by Eq. (11). *Q* and *Q*_*M*_ are the actual volume transfering rate and the theoretical maximum volume transfering rate of the screw conveyor, respectively.

11 $$\theta =\arctan \left[\tan \,\varphi \,{\left(\frac{Qm}{Q}-1\right)}^{-1}\right]$$

According to the stress analysis of the soil microelement in the spiral tube, the mechanical equilibrium Eq. (12) in the direction of the spiral axis can be obtained.

12 $$d{({f}_{m})}_{l}\cdot d\cdot {Q}_{n}\cdot \,\cos \,{\varphi }_{m}=(\begin{array}{c}{\tau }_{c}\cdot {({c}_{t})}_{w}\cdot d{({f}_{t})}_{l}\cdot \,\sin \,\theta +{\tau }_{s}\cdot {({c}_{b})}_{w}\cdot d{({f}_{b})}_{l}\cdot \,\sin \,{\varphi }_{s}\\ +{({\tau }_{f})}_{t}\cdot d\cdot d{({f}_{m})}_{l}\cdot \,\sin \,{\varphi }_{m}+{({\tau }_{f})}_{b}\cdot d\cdot d{({f}_{m})}_{l}\cdot \,\sin \,{\varphi }_{m}\\ +{({c}_{m})}_{w}\cdot d\cdot dP\cdot \,\sin \,{\varphi }_{m}+{({c}_{m})}_{w}\cdot d\cdot \rho .g\cdot d{({f}_{m})}_{l}\cdot \,\sin \,\phi \end{array})$$

Where*ρ* is the density of the conditioned soil, and *g* is the gravitational acceleration, taking 9.8 N/kg.

### Load torque equilibrium equation when rotating along the screw axis

According to the load torque equilibrium condition of the conditioned soil in the spiral pipe rotating along the axis during the steady state construction of the shield screw conveyor, the Eq. (13) can be obtained.

13 $$d{({f}_{m})}_{l}\cdot d\cdot {Q}_{n}\cdot \,\sin \,{\varphi }_{m}\cdot \left(R,-,\frac{d}{2}\right)=\left(\begin{array}{c}{\tau }_{c}\cdot {({c}_{t})}_{w}\cdot d{({f}_{t})}_{l}\cdot \,\cos \,\theta \cdot R\\ -{\tau }_{s}\cdot {({c}_{b})}_{w}\cdot d{({f}_{b})}_{l}\cdot \,\cos \,{\varphi }_{s}\cdot (R-d)\\ -{({\tau }_{f})}_{t}\cdot d\cdot d{({f}_{m})}_{l}\cdot \,\cos \,{\varphi }_{m}\cdot (R-\frac{d}{2})\\ -{({\tau }_{f})}_{b}\cdot d\cdot d{({f}_{m})}_{l}\cdot \,\cos \,{\varphi }_{m}\cdot (R-\frac{d}{2})\\ -{({c}_{m})}_{w}\cdot d\cdot dP\cdot \,\cos \,{\varphi }_{m}\cdot (R-\frac{d}{2})\\ -{({c}_{m})}_{w}\cdot d\cdot d{({f}_{m})}_{l}\cdot \rho \cdot g\cdot \,\cos \,\phi \cdot \left(R-\frac{d}{2}\right)\end{array}\right)$$

### Calculation of axial pressure distribution of screw conveyor

Divide both sides of the equation in Eq. (12) by d (*f*_*m*_)_*l*_ to get Eq. (14):

14 $$d\cdot {Q}_{n}\cdot \,\cos \,{\varphi }_{m}=\left(\begin{array}{c}{\tau }_{c}\cdot {({c}_{t})}_{w}\cdot \frac{d{({f}_{t})}_{l}}{d{({f}_{m})}_{l}}\cdot \,\sin \,\theta +{\tau }_{s}\cdot {({c}_{b})}_{w}\cdot \frac{d{({f}_{b})}_{l}}{d{({f}_{m})}_{l}}\cdot \,\sin \,{\varphi }_{s}\\ +{({\tau }_{f})}_{t}\cdot d\cdot \,\sin \,{\varphi }_{m}+{({\tau }_{f})}_{b}\cdot d\cdot \,\sin \,{\varphi }_{m}\\ +\frac{{({c}_{m})}_{w}\cdot d\cdot dP\cdot \,\sin \,{\varphi }_{m}}{d{({f}_{m})}_{l}}+{({c}_{m})}_{w}\cdot d\cdot \rho .g\cdot \,\sin \,\phi \end{array}\right)$$

For Eq. (13), divide both sides of the equation by d(*f*_*m*_)_*l*_ and $(R-\frac{d}{2})$ to get Eq. (15):

15 $$d\cdot {Q}_{n}\cdot \,\sin \,{\varphi }_{m}=\left(\begin{array}{c}{\tau }_{c}\cdot {({c}_{t})}_{w}\cdot \frac{d{({f}_{t})}_{l}}{d{({f}_{m})}_{l}}\cdot \,\cos \,\theta \cdot \frac{R}{\left(R,-,\frac{d}{2}\right)}\\ -{\tau }_{s}\cdot {({c}_{b})}_{w}\cdot \frac{d{({f}_{b})}_{l}}{d{({f}_{m})}_{l}}\cdot \,\cos \,{\varphi }_{s}\cdot \frac{(R-d)}{\left(R,-,\frac{d}{2}\right)}\\ -{({\tau }_{f})}_{t}\cdot d\cdot \,\cos \,{\varphi }_{m}\\ -{({\tau }_{f})}_{b}\cdot d\cdot \,\cos \,{\varphi }_{m}\\ -\frac{{({c}_{m})}_{w}\cdot d\cdot dP\cdot \,\cos \,{\varphi }_{m}}{d{({f}_{m})}_{l}}\\ -{({c}_{m})}_{w}\cdot d\cdot \rho \cdot g\cdot \,\cos \,\phi \end{array}\right)$$

According to the relationship between the size of the study object and the parameters of the spiral structure, the following relational expressions are derived and made constants *k*_*1*_ and *k*_*2*_, respectively. Details as follows:

16 $$\frac{d{({f}_{t})}_{l}}{d{({f}_{m})}_{l}}=\frac{R}{R-\frac{d}{2}}={k}_{1}$$

17 $$\frac{d{({f}_{b})}_{l}}{d{({f}_{m})}_{l}}=\frac{R-d}{R-\frac{d}{2}}={k}_{2}$$

Substituting Eq. (16) and Eq. (17) into Eq. (14) and Eq. (15), Eq. (18) and Eq. (19) are obtained, respectively.

18 $$d\cdot {Q}_{n}\cdot \,\cos \,{\varphi }_{m}=\left(\begin{array}{c}{\tau }_{c}\cdot {({c}_{t})}_{w}\cdot {k}_{1}\cdot \,\sin \,\theta +{\tau }_{s}\cdot {({c}_{b})}_{w}\cdot {k}_{2}\cdot \,\sin \,{\varphi }_{s}\\ +{({\tau }_{f})}_{t}\cdot d\cdot \,\sin \,{\varphi }_{m}+{({\tau }_{f})}_{b}\cdot d\cdot \,\sin \,{\varphi }_{m}\\ +\frac{{({c}_{m})}_{w}\cdot d\cdot dP\cdot \,\sin \,{\varphi }_{m}}{d{({f}_{m})}_{l}}+{({c}_{m})}_{w}\cdot d\cdot \rho .g\cdot \,\sin \,\phi \end{array}\right)$$

19 $$d\cdot {Q}_{n}\cdot \,\sin \,{\varphi }_{m}=\left(\begin{array}{c}{\tau }_{c}\cdot {({c}_{t})}_{w}\cdot \,\cos \,\theta \cdot {k}_{1}^{2}\\ -{\tau }_{s}\cdot {({c}_{b})}_{w}\cdot \,\cos \,{\varphi }_{s}\cdot {k}_{2}^{2}\\ -{({\tau }_{f})}_{t}\cdot d\cdot \,\cos \,{\varphi }_{m}\\ -{({\tau }_{f})}_{b}\cdot d\cdot \,\cos \,{\varphi }_{m}\\ -\frac{{({c}_{m})}_{w}\cdot d\cdot dP\cdot \,\cos \,{\varphi }_{m}}{d{({f}_{m})}_{l}}\\ -{({c}_{m})}_{w}\cdot d\cdot \rho \cdot g\cdot \,\cos \,\phi \end{array}\right)$$

Both sides of the Eq. (18) divide both sides of the Eq. (19) to get Eq. (20), as follows:

20 $$\frac{\cos \,{\varphi }_{m}}{\sin \,{\varphi }_{m}}=\frac{\left(\begin{array}{c}{\tau }_{c}\cdot {({c}_{t})}_{w}\cdot {k}_{1}\cdot \,\sin \,\theta +{\tau }_{s}\cdot {({c}_{b})}_{w}\cdot {k}_{2}\cdot \,\sin \,{\varphi }_{s}+{({\tau }_{f})}_{t}\cdot d\cdot \,\sin \,{\varphi }_{m}\\ +{({\tau }_{f})}_{b}\cdot d\cdot \,\sin \,{\varphi }_{m}+\frac{{({c}_{m})}_{w}\cdot d\cdot dP\cdot \,\sin \,{\varphi }_{m}}{d{({f}_{m})}_{l}}+{{(c)}_{m}}_{w}\cdot d\cdot \rho .g\cdot \,\sin \,\phi \end{array}\right)}{\left(\begin{array}{c}{\tau }_{c}\cdot {({c}_{t})}_{w}\cdot \,\cos \,\theta \cdot {k}_{1}^{2}-{\tau }_{s}\cdot {({c}_{b})}_{w}\cdot \,\cos \,{\varphi }_{s}\cdot {k}_{2}^{2}-{({\tau }_{f})}_{t}\cdot d\cdot \,\cos \,{\varphi }_{m}\\ -{({\tau }_{f})}_{b}\cdot d\cdot \,\cos \,{\varphi }_{m}-\frac{{({c}_{m})}_{w}\cdot d\cdot dP\cdot \,\cos \,{\varphi }_{m}}{d{({f}_{m})}_{l}}-{({c}_{m})}_{w}\cdot d\cdot \rho \cdot g\cdot \,\cos \,\phi \end{array}\right)}$$

Multiply the fractional expressions on both sides of the Eq. (20) to get Eq. (21), as follows:

21 $$\begin{array}{c}{\tau }_{c}\cdot {({c}_{t})}_{w}\cdot {k}_{1}^{2}\cdot \,\cos \,\theta \cdot \,\cos \,{\varphi }_{m}-{\tau }_{s}\cdot {({c}_{b})}_{w}\cdot {k}_{2}^{2}\cdot \,\cos \,{\varphi }_{s}\cdot \,\cos \,{\varphi }_{m}-[{({\tau }_{f})}_{t}+{({\tau }_{f})}_{b}]\cdot d\cdot {(\cos {\varphi }_{m})}^{2}\\ -\,\frac{{({c}_{m})}_{w}\cdot d\cdot dP\cdot {(\cos {\varphi }_{m})}^{2}}{d{({f}_{m})}_{l}}-{({c}_{m})}_{w}\cdot d\cdot \rho \cdot g\cdot \,\cos \,\phi \cdot \,\cos \,{\varphi }_{m}\\ =\,{\tau }_{c}\cdot {({c}_{t})}_{w}\cdot {k}_{1}\cdot \,\sin \,\theta \cdot \,\sin \,{\varphi }_{m}+{\tau }_{s}\cdot {({c}_{b})}_{w}\cdot {k}_{2}\cdot \,\sin \,{\varphi }_{s}\cdot \,\sin \,{\varphi }_{m}+[{({\tau }_{f})}_{t}+{({\tau }_{f})}_{b}]\cdot d\cdot {(\sin {\varphi }_{m})}^{2}\\ +\,\frac{{({c}_{m})}_{w}\cdot d\cdot dP\cdot {(\sin {\varphi }_{m})}^{2}}{d{({f}_{m})}_{l}}+{({c}_{m})}_{w}\cdot d\cdot \rho \cdot g\cdot \,\sin \,\phi \cdot \,\sin \,{\varphi }_{m}\end{array}$$

Sorting the d*P* item in Eq. (21) to the left of the equation gives Eq. (22), as shown in the following:

22 $$dP=\frac{1}{d}\cdot \left\{\begin{array}{c}{\tau }_{c}\cdot \frac{{({c}_{t})}_{w}}{{({c}_{m})}_{w}}\cdot [{k}_{1}^{2}\cdot \,\cos \,\theta \cdot \,\cos \,{\varphi }_{m}-{k}_{1}\cdot \,\sin \,\theta \cdot \,\sin \,{\varphi }_{m}]\\ -{\tau }_{s}\cdot \frac{{({c}_{b})}_{w}}{{({c}_{m})}_{w}}\cdot [{k}_{2}^{2}\cdot \,\cos \,{\varphi }_{s}\cdot \,\cos \,{\varphi }_{m}+{k}_{2}\cdot \,\sin \,{\varphi }_{s}\cdot \,\sin \,{\varphi }_{m}]\\ -\frac{[{({\tau }_{f})}_{t}+{({\tau }_{f})}_{b}]\cdot d}{{({c}_{m})}_{w}}-d\cdot \rho \cdot g\cdot [\cos \,\phi \cdot \,\cos \,{\varphi }_{m}+\,\sin \,\phi \cdot \,\sin \,{\varphi }_{m}]\end{array}\right\}\cdot d{({f}_{m})}_{l}$$

In the above equation, d*P* is the pressure change in the spiral groove direction. According to the expression (5) of the spiral groove direction and the spiral axis direction, substituting Eq. (5) into Eq. (22) can derive the pressure gradient differential equation along the spiral axis, as shown in Eq. (23).

23 $$d{P}_{y}={\sin }^{-1}{\varphi }_{m}\cdot \frac{1}{d}\cdot \left\{\begin{array}{c}{\tau }_{c}\cdot \frac{{({c}_{t})}_{w}}{{({c}_{m})}_{w}}\cdot [{k}_{1}^{2}\cdot \,\cos \,\theta \cdot \,\cos \,{\varphi }_{m}-{k}_{1}\cdot \,\sin \,\theta \cdot \,\sin \,{\varphi }_{m}]\\ -{\tau }_{s}\cdot \frac{{({c}_{b})}_{w}}{{({c}_{m})}_{w}}\cdot [{k}_{2}^{2}\cdot \,\cos \,{\varphi }_{s}\cdot \,\cos \,{\varphi }_{m}+{k}_{2}\cdot \,\sin \,{\varphi }_{s}\cdot \,\sin \,{\varphi }_{m}]\\ -\frac{[{({\tau }_{f})}_{t}+{({\tau }_{f})}_{b}]\cdot d}{{({c}_{m})}_{w}}-d\cdot \rho \cdot g\cdot [\cos \,\phi \cdot \,\cos \,{\varphi }_{m}+\,\sin \,\phi \cdot \,\sin \,{\varphi }_{m}]\end{array}\right\}\cdot dy$$

According to the relational expressions (8) to (10) between the spiral groove width and the spiral structure parameters (spiral pitch, spiral blade thickness, and spiral angle at different positions), substitute them into Eq. (23) to get Eq. (24).

24 $$d{P}_{y}={\sin }^{-1}{\varphi }_{m}\cdot \frac{1}{d}\cdot \left\{\begin{array}{c}{\tau }_{c}\cdot \frac{\cos \,\varphi }{\cos \,{\varphi }_{m}}\cdot [{k}_{1}^{2}\cdot \,\cos \,\theta \cdot \,\cos \,{\varphi }_{m}-{k}_{1}\cdot \,\sin \,\theta \cdot \,\sin \,{\varphi }_{m}]\\ -{\tau }_{s}\cdot \frac{\cos \,{\varphi }_{s}}{\cos \,{\varphi }_{m}}\cdot [{k}_{2}^{2}\cdot \,\cos \,{\varphi }_{s}\cdot \,\cos \,{\varphi }_{m}+{k}_{2}\cdot \,\sin \,{\varphi }_{s}\cdot \,\sin \,{\varphi }_{m}]\\ -\frac{[{({\tau }_{f})}_{t}+{({\tau }_{f})}_{b}]\cdot d}{(s-e)\cdot \,\cos \,{\varphi }_{m}}-d\cdot \rho \cdot g\cdot [\cos \,\phi \cdot \,\cos \,{\varphi }_{m}+\,\sin \,\phi \cdot \,\sin \,{\varphi }_{m}]\end{array}\right\}\cdot dy$$

Further arranging Eq. (24), we can get the Eq. (25) for calculating the pressure gradient along the spiral axis of the shield screw conveyor.

25 $$d{P}_{y}=\frac{1}{d}\cdot \left\{\begin{array}{c}-{k}_{1}\cdot {\tau }_{c}\cdot \left[\frac{\cos \,\varphi }{\cos \,{\varphi }_{m}}\cdot \,\sin \,\theta -{k}_{1}\cdot \frac{\cos \,\theta \cdot \,\cos \,\varphi }{\sin \,{\varphi }_{m}}\right]\\ -{k}_{2}\cdot {\tau }_{s}\cdot \left[{k}_{2}\cdot \frac{{(\cos {\varphi }_{s})}^{2}}{\sin \,{\varphi }_{m}}+\frac{\cos \,{\varphi }_{s}}{\cos \,{\varphi }_{m}}\cdot \,\sin \,{\varphi }_{s}\right]\\ -\frac{[{({\tau }_{f})}_{t}+{({\tau }_{f})}_{b}]\cdot d}{(s-e)\cdot \,\cos \,{\varphi }_{m}\cdot \,\sin \,{\varphi }_{m}}-d\cdot \rho \cdot g\cdot \left[\frac{\cos \,\phi \cdot \,\cos \,{\varphi }_{m}}{\sin \,{\varphi }_{m}}+\,\sin \,\phi \right]\end{array}\right\}\cdot dy$$

After Eq. (25) is integrated along the spiral axis of length *L*, the Eq. (26) for calculating the pressure distribution along the spiral axis of the shield screw conveyor can be obtained.

26 $${P}_{y}=\frac{y}{d}\cdot \left\{\begin{array}{c}-{k}_{1}\cdot {\tau }_{c}\cdot \left[\frac{\cos \,\varphi }{\cos \,{\varphi }_{m}}\cdot \,\sin \,\theta -{k}_{1}\cdot \frac{\cos \,\theta \cdot \,\cos \,\varphi }{\sin \,{\varphi }_{m}}\right]\\ -{k}_{2}\cdot {\tau }_{s}\cdot \left[{k}_{2}\cdot \frac{{(\cos {\varphi }_{s})}^{2}}{\sin \,{\varphi }_{m}}+\frac{\cos \,{\varphi }_{s}}{\cos \,{\varphi }_{m}}\cdot \,\sin \,{\varphi }_{s}\right]\\ -\frac{[{({\tau }_{f})}_{t}+{({\tau }_{f})}_{b}]\cdot d}{(s-e)\cdot \,\cos \,{\varphi }_{m}\cdot \,\sin \,{\varphi }_{m}}-d\cdot \rho \cdot g\cdot \left[\frac{\cos \,\phi \cdot \,\cos \,{\varphi }_{m}}{\sin \,{\varphi }_{m}}+\,\sin \,\phi \right]\end{array}\right\}+{k}_{0}$$

Where 0 ≤ *y* ≤ *L*.

Based on the initial condition *L* = 0, *P*_*y*_ = *P*_*0*_, the Eq. (26) can be equivalent to Eq. (27).

27 $${P}_{y}=\frac{y}{d}\cdot \left\{\begin{array}{c}-{k}_{1}\cdot {\tau }_{c}\cdot \left[\frac{\cos \,\varphi }{\cos \,{\varphi }_{m}}\cdot \,\sin \,\theta -{k}_{1}\cdot \frac{\cos \,\theta \cdot \,\cos \,\varphi }{\sin \,{\varphi }_{m}}\right]\\ -{k}_{2}\cdot {\tau }_{s}\cdot \left[{k}_{2}\cdot \frac{{(\cos {\varphi }_{s})}^{2}}{\sin \,{\varphi }_{m}}+\frac{\cos \,{\varphi }_{s}}{\cos \,{\varphi }_{m}}\cdot \,\sin \,{\varphi }_{s}\right]\\ -\frac{[{({\tau }_{f})}_{t}+{({\tau }_{f})}_{b}]\cdot d}{(s-e)\cdot \,\cos \,{\varphi }_{m}\cdot \,\sin \,{\varphi }_{m}}-d\cdot \rho \cdot g\cdot \left[\frac{\cos \,\phi \cdot \,\cos \,{\varphi }_{m}}{\sin \,{\varphi }_{m}}+\,\sin \,\phi \right]\end{array}\right\}+{P}_{0}$$

The above equation shows that under the conditions of the helical structure and the installation leaning angle, the parameters *k*_*1*_, *k*_*2*_, *cosφ*, *cosφ*_*m*_, *sinφ*_*m*_, *cosφ*_*s*_, *sinφ*_*s*_, *s*, *e*, *d*, *φ*, *g*, *ϕ* are constants, and the parameter *cosθ* is a variable which changes with the change of the rotation speed of the screw shaft, that is, the conveying angle of the soil flow changes with the rotation speed of the motor, which causes the change of the volume transfer rate of the soil. When the rotation speed of the screw conveyor is constant, the shear stress between the conditioned soil and the spiral structure of the shield screw conveyor is constant, the conclusion that the pressure field of the shield screw conveyor is distributed along the spiral axis as a linear decreasing function of the spiral length *y* can be derived.

## Laboratory tests

**Figure 5:**
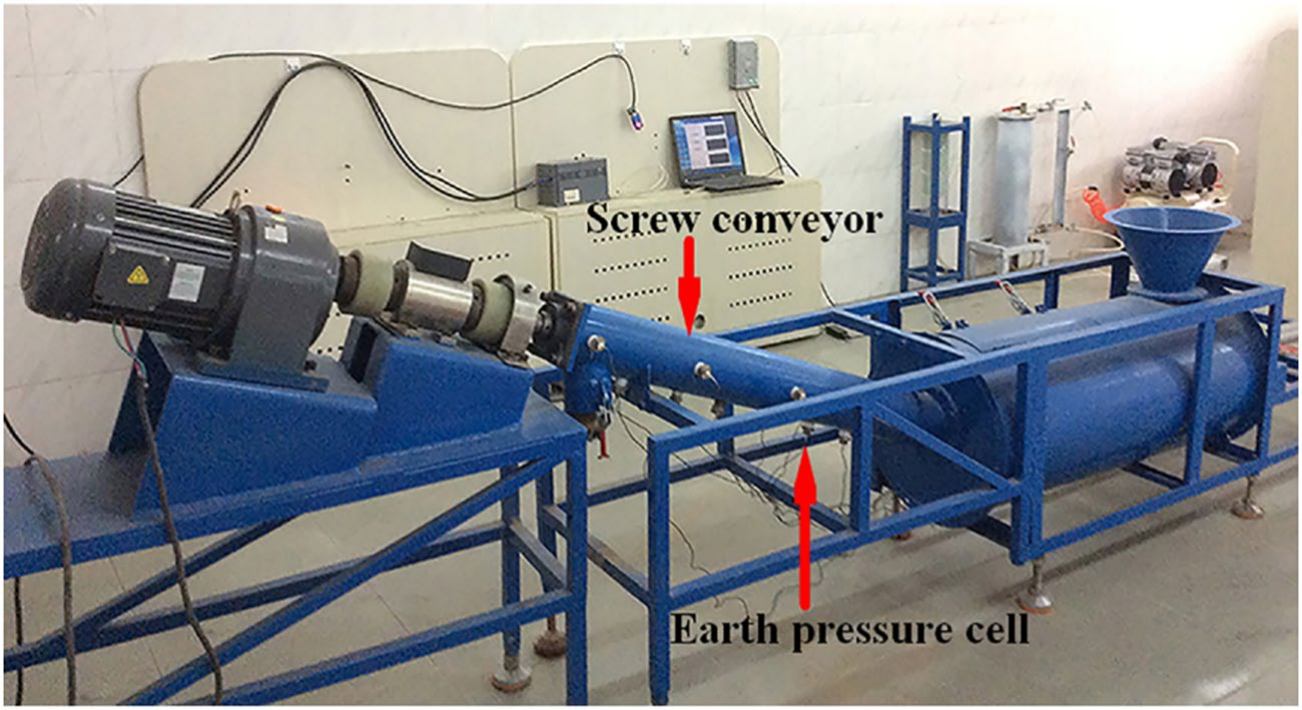
Shield model screw conveyor.

**Figure 6:**
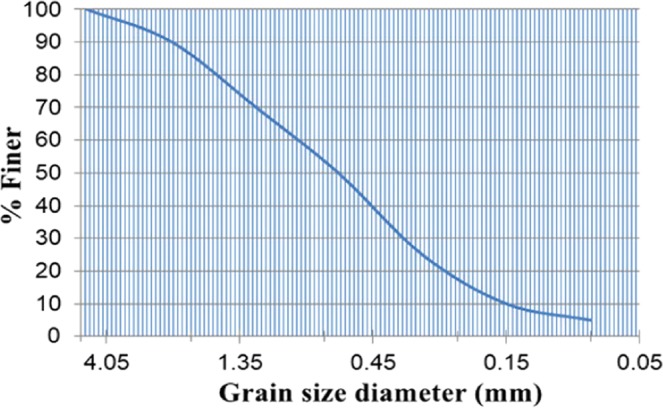
Grain size distribution of the standard sand.

**Figure 7:**
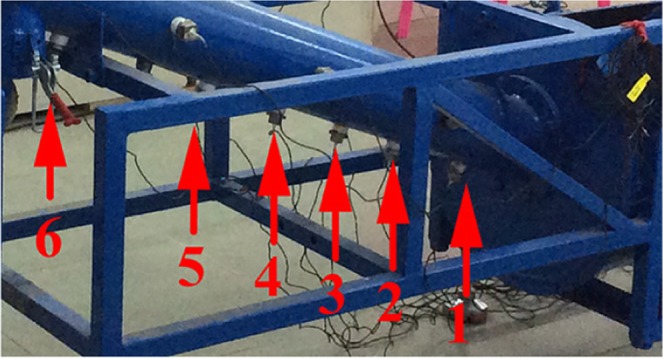
Earth pressure cells installed (Pointed by the red arrows).

According to the parameters of the earth pressure balanced shield screw conveyor used in Beijing Metro Line 14, the company was commissioned to process the scale shield screw conveyor at a ratio of 1:10, as shown in Fig. 5^36^. The experiment material is the ISO stand sand manufactured by Xiamen Iso Standard Sand Co, Ltd. The particle size distribution is shown in the Fig. 6. The sandy soil conditioning schemes used are a concentration of 7% foaming agent solution with a blending ratio of 10% and a water-soil quality of 5:1 sodium-based bentonite slurry with a blending ratio of 15%. In order to measure the pressure field distribution of the conditioned soil in the spiral tube, six earth pressure celles are arranged on the spiral tube shell of the model machine, and its installation position is shown in Fig. 7.

The normal compressive stress affects the magnitude of the friction coefficient. The reduction of normal stress can reduce the friction coefficient slightly. A smaller coefficient of friction will reduce the torque required to extract the conditioned soil from the pressurized chamber along the screw conveyor in the earth pressure balanced shield machine, and it will also help to improve the spiral shaft, spiral blades and spiral shell wear problems. Therefore, studying the stress changes of the spiral tube of the model machine under different operating conditions will help to better understand the effect of foam conditioning agent on the characteristics of soil and the change of the earth pressure balanced shield screw speed on its working performance.

### Spiral tube pressure field distribution when the motor speed is 6 rpm

**Figure 8:**
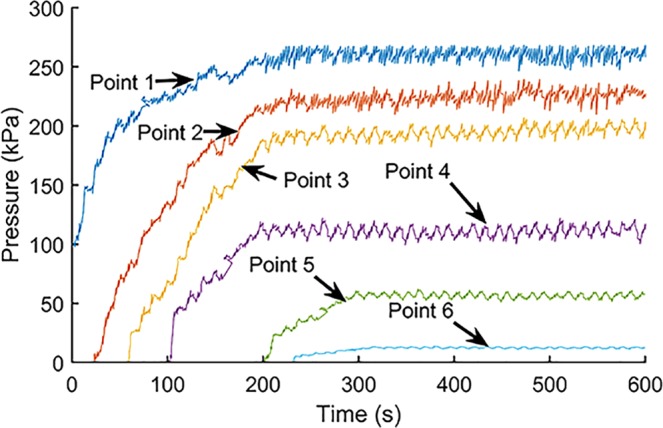
Compressive stresses at 6 rpm.

When the motor speed is 6 rpm, the indoor test results are shown in Fig. 8. The normal stress values shown in the Figures have similar orders of magnitude, which is the normal case for all tests performed on a model screw conveyor.

Looking at the change of the compressive stress at each measuring point of the spiral tube with time, it can be seen that the compressive stress at each measuring point enters the steady state interval after a certain rise time. Comparing the change of compressive stress at each measuring point, the compressive stress at measuring point 1 is larger than other measuring results. This is because the measuring point 1 is close to the soil sample box, so the compressive stress is larger than that at other measuring points. On the whole, the measuring results of compressive stress at each measuring point gradually change from the measurement point 1 near the soil bin to the measurement point 6 near the soil outlet.

### Spiral tube pressure field distribution when the motor speed is 16 rpm

**Figure 9:**
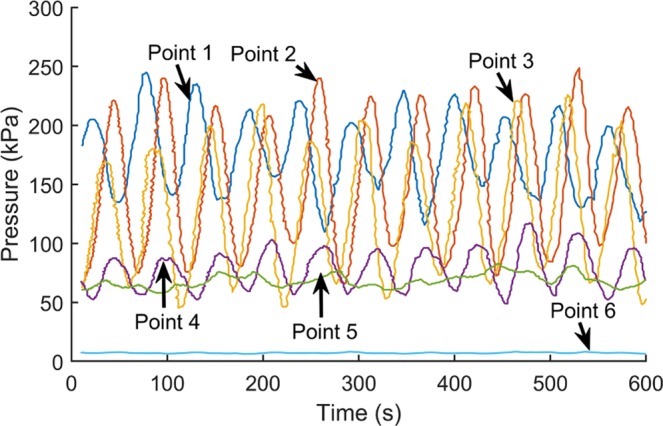
Compressive stresses at 16 rpm.

In order to study the influence of the increase of the motor speed on the spiral tube pressure distribution, the compressive stress distribution of the spiral tube when the motor speed is 16 rpm is shown below, see Fig. 9. It should be noted that the start time of the inspection at this speed starts after 10 seconds.

Comparing the time-history curves under different operating conditions, it can be seen that after the motor speed is increased from 6 rpm to 16 rpm, the change of the compressive stress at each measurement point along the spiral direction with time is similar to the pressure distribution when the motor is rotating at low speed. At the same time, the pressure values at the corresponding measuring points have decreased in magnitude. Engineering practice shows that the rotation speed of the screw conveyor has a certain effect on the operation of the earth pressure balance shield machine, and the increase in the speed of the motor can accelerate the pressure dissipation in the shield chamber.

### Pressure gradient of the model screw conveyor at steady state

**Figure 10:**
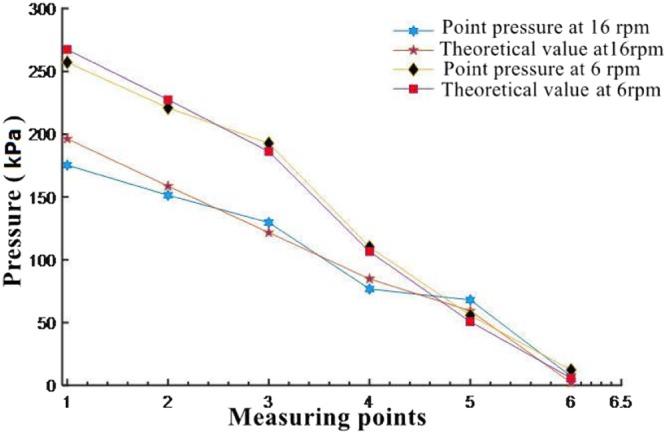
Screw conveyor pressure gradient at different speeds.

When the motor speed is 6 rpm and 16 rpm, respectively. Figure 10 shows the pressure gradient distribution of each measuring point along the direction of the spiral tube when the model machine runs for 400 seconds.

The measuring values and theoretical calculation results of each measuring point of the spiral tube are shown in the above figure. It can be seen that the theoretical value is in good agreement with the actual measuring result. The maximum relative error of the screw shaft at 6 rpm is 4.2%. When the rotation speed of the screw shaft is 16 rpm, the relative error between the measurement result and the theoretical value is 10.7%. The pressure distribution of each measuring point of the spiral tube at different speeds shows that the pressure distribution from the measuring point near the soil box to the measuring point in the direction of the soil outlet shows a decreasing trend. At the same time, it can be seen that the linearity of the pressure gradient at low speed is better than that at high speed.

## Conclusions

By analyzing the stress of the conditioned soil in the spiral tube, a theoretical calculation model describing the pressure field distribution and pressure gradient of the earth pressure balanced shield screw conveyor was proposed.

(1) The pressure field distribution is related to the spiral geometry, the rotation speed of the spiral, the soil flow conveying angle, and the mechanical properties of the soil after adding the conditioning agent. The standard sand produced by Xiamen ISO Co., Ltd. was used to carry out the indoor model screw conveyor tests, which verified the validity of the proposed theoretical calculation model. This test accurately simulates the key parameters that affect the operation of the full-size screw conveyor in the earth pressure balanced shield tunneling machine (the rotation speed of the screw conveyor). The test results are directly related to the screw conveyors of different sizes (ie, the measuring results are affected by the spiral structure parameters).

(2) When the appropriate parameters are input (that is, the conditioned soil is a plastic fluid and the shear stress is the constant), the proposed theoretical model can accurately predict the linear distribution of the pressure field gradient along the spiral direction. Whether the pressure field gradient is increasing or decreasing is controlled by the shear stress of the conditioned soil. The pressure gradient of the plastic fluid-like conditioned soil along the spiral from the soil inlet to the soil outlet will decrease linearly; When the conditioned soil is too hard, that is, the shear stress between the conditioned soil and the spiral structure is too large, the screw conveyor will become clogged, and the pressure field shows an increasing trend along the spiral direction.

(3) The shield screw conveyor in the project is provided with front and rear gates. The influence of the opening of the gate on the pressure field distribution of the conditioned soil in the spiral pipe is not considered in the theoretical model and the model screw conveyor. This is also one of the contents to be considered in the next research work.

(4) The conditioned soil pressure field distribution and pressure gradient calculation method proposed are derived on the assumption that the soil filling rate is 100% and the conditioned soil is a homogeneous plastic fluid. At the same time, for simplicity and practicality, the shear stresses between the soil and the screw structure are equivalent to a parameter, and it is assumed that the shear stresses along the helical axis remain the same. As for the true variation of the shear stresses around the helical structure, it is still unknown.

## Data Availability

The data generated during the current study are available from the corresponding author on reasonable request.
